# Deep Transcriptomic Analysis of Black Rockfish (*Sebastes schlegelii*) Provides New Insights on Responses to Acute Temperature Stress

**DOI:** 10.1038/s41598-018-27013-z

**Published:** 2018-06-14

**Authors:** Likang Lyu, Haishen Wen, Yun Li, Jifang Li, Ji Zhao, Simin Zhang, Min Song, Xiaojie Wang

**Affiliations:** The Key Laboratory of Mariculture (Ocean University of China), Ministry of Education, Ocean University of China, Qingdao, P.R. China

## Abstract

In the present study, we conducted an RNA-Seq analysis to characterize the genes and pathways involved in acute thermal and cold stress responses in the liver of black rockfish, a viviparous teleost that has the ability to cope with a wide range of temperature changes. A total of 584 annotated differentially expressed genes (DEGs) were identified in all three comparisons (HT vs NT, HT vs LT and LT vs NT). Based on an enrichment analysis, DEGs with a potential role in stress accommodation were classified into several categories, including protein folding, metabolism, immune response, signal transduction, molecule transport, membrane, and cell proliferation/apoptosis. Considering that thermal stress has a greater effect than cold stress in black rockfish, 24 shared DEGs in the intersection of the HT vs LT and HT vs NT groups were enriched in 2 oxidation-related gene ontology (GO) terms. Nine important heat-stress-reducing pathways were significantly identified and classified into 3 classes: immune and infectious diseases, organismal immune system and endocrine system. Eight DEGs (*early growth response protein 1, bile salt export pump*, *abcb11, hsp70a, rtp3, 1,25-dihydroxyvitamin d(3) 24-hydroxylase, apoa4, transcription factor jun-b-like* and an uncharacterized gene) were observed among all three comparisons, strongly implying their potentially important roles in temperature stress responses.

## Introduction

Ecosystems are currently exposed to global warming and climate change. One of the most direct impacts of climate change on the marine ecosystem affects fisheries. It has been reported that the temperature of the upper ocean (0 to 700 m depth) has increased, rising with an average rate of 0.05 °C per decade since 1971. The rate of temperature change is highest near the surface of the ocean (>0.1 °C per decade in the upper 75 m from 1971 to 2010)^1^. Fish are poikilothermic aquatic animals whose body temperatures adapt to environmental temperatures to a certain degree, changes in water temperatures may affect their growth, survival, reproduction, development and physiological performances^2,3^.

The molecular mechanisms underlying temperature stress conditions have long been of interest. Temperature stress causes expression changes in a series of stress-responsive genes, such as genes regulating protein folding repair^4,5^, energy metabolism^6,7^, the oxidation reduction process^7^, and the control of the cell cycle^8,9^. The identification of stress-responsive genes and pathways is the first step to reveal the fundamental mechanisms of the response to thermal stress and to predict the capacity of fish to adapt to climate changes. The next-generation sequencing technology (NGS)-based RNA-Seq platform is considered to be a revolutionary and efficient tool for investigating stress-responsive genes, as it can quantify over millions of unknown transcripts at once. RNA-Seq has been applied in studies of responses to temperature stress in several fish species, such as catfish^7^, Australian rainbowfish^10^, and snow trout^11^. However, almost all of these studies focused on oviparous fish species.

Ovoviviparity is a unique fish reproduction mode, in which fertilized eggs cannot delivered from the female ovary until the embryos are mature. Black rockfish (*Sebastes schlegelii)*, belonging to Scorpaenidae, is an economically important marine ovoviviparous teleost, which is widely distributed in Japan, Korea, and northeast coast of China. Black rockfish can survive temperatures ranging from 5 °C to 28 °C, with the optimal temperature ranging from 18 °C to 24 °C^12^. In the current environment, black rockfish experience serious acute temperature stress which may cause heat shock, disease, and metabolic problems, especially reproduction problems. Previous studies on temperature stress in black rockfish have focused on the measurement of basic physiological and biochemical indexes^13–15^ or the cloning and expression level detection of a few stress-related genes^16^. However, little is known about the molecular mechanisms underlying temperature adaptation and thermal stress response in black rockfish. In this study, RNA-Seq was performed on liver samples to characterize genes and pathways involved in temperature stress response in black rockfish. Without a reference genome, the transcripts were *de novo* assembled and annotated, which greatly enriched the gene database for black rockfish. The temperature stress-induced genes identified in this study also provide a valuable candidate gene list for the establishment of heat- or cold-resistant fish lines.

## Results

### Raw sequencing data and *de novo* assembly

RNA-Seq was performed on liver samples from three different temperature treatment groups (HT, LT, NT). A total of 404,780,554 raw reads (150 bp) were obtained from 9 liver samples on the Illumina HiSeq. 4000 platform. After preprocessing and the filtration of low-quality sequences, the clean read count was 390,616,892(Table 1).

**Table 1:** Summary of statistics for Illumina short reads of the liver transcriptome of black rockfish.

Sample^a^	Raw Reads	Clean Reads	Q20(%)^b^	Q30(%)^c^	Clean Bases	Total Mapped (%)^d^
HT_1	80,907,966	78,190,600	98.29	95.89	11.73G	65,668,692(83.99%)
HT_2	69,242,838	66,727,542	97.98	95.37	10.01G	53,962,070(80.87%)
LT_1	73,014,860	70,622,792	98.28	95.88	10.59G	58,845,154(83.32%)
LT_2	65,781,362	63,264,454	97.99	95.51	9.49G	49,981,194(79.00%)
NT_1	51,940,444	50,153,552	98.26	95.93	7.52G	40,858,960(81.47%)
NT_2	63,893,084	61,657,952	98.32	95.99	9.25G	50,719,810(82.26%)
Total	404,780,554	390,616,892				

^a^1 and 2: Two independent biological replicates;

^b^Q20: The percentage of bases with a Phred value > 20;

^c^Q30: The percentage of bases with a Phred value > 30;

^d^The number of clean reads that mapped onto the assembled reference transcriptome.

After the *de novo* assembly analysis based on all the Illumina clean reads, a total of 250,326 transcripts were generated (Table 2). These transcripts ranged from 201 to 16,112 bp in length, with an N50 length of 880 bp.

**Table 2:** Summary of assembly and annotation statistics of the liver transcriptome of black rockfish.

Category	Number of transcripts
Total number of clean reads of NT	111,811,504
Total number of clean reads of LT	133,887,246
Total number of clean reads of HT	144,918,142
Average length of all transcripts (bp)	589
N50 length of all transcripts (bp)	880
Max length (bp)	16,112
Min length (bp)	201
Total number of annotated transcripts in the Nr database	66,596 (30.7%)
Total number of annotated transcripts in the Nt database	97,200 (44.8%)
Total number of annotated transcripts in the KEGG database	52,176 (24.05%)
Total number of annotated transcripts in the Swiss-Prot database	54,751 (25.24%)
Total number of annotated transcripts in the PFAM database	46,426 (21.4%)
Total number of annotated transcripts in the GO database	47427 (21.86%)
Total number of annotated transcripts in the KOG database	29,206 (13.46%)
Total number of annotated transcripts in at least one database	109,302 (50.38%)

### Annotation and function analysis of liver transcripts

All transcripts were subjected to annotation analysis by a comparison with the Nr, Nt, KEGG, KO, Swiss-Prot, PFAM, GO and KOG database. The results in Table 2 show the number of annotated transcripts in each database. A total number of 109,302(50.38%) transcripts were annotated by at least one database, and 66,596 (50.38%) annotated transcripts showed a significant BLAST hit against Nr database.

For the 66,596 transcripts that matched against the Nr database, the most abundant BLAST hits were from fish species (35.5%) such as *Larimichthys crocea* (19%), *Stegastes partitus* (8.9%) and *Notothenia coriiceps* (7.6%), followed by some other species, *Homo sapiens* (11.3%), *Schistosoma japonicum* (8.7%)and others (44.5%) (Supplement 1a).

The functional classification of transcriptome data is the primary requirement for the application of functional genomic approaches in fishery research. GO and KEGG analyses are currently the most popular methods used for the functional classification of transcriptomic sequences. Our results showed that Blast2Go assigned 47,427 transcripts 56 functional GO terms (Supplement 1b). Regarding the three primary ontology categories, BP represents the majority (25 terms) of annotations, followed by CC (20 terms) and MF (11 terms). Based on the analysis of level 2 GO terms, the GO terms in BP with the highest numbers of annotations were cellular process (GO:0009987), metabolic process (GO:0008152), single-organism process (GO:0044699), biological regulation (GO:0065007) and regulation of biological process (GO:0050789). For CC, cell (GO:0005623), cell part (GO:0044464), organelle (GO:0043226) and macromolecular complex (GO:0032991) contained the highest numbers of annotations. The GO terms related to MF with the highest number of annotations were binding (GO:0005488), catalytic activity (GO:0003824) and transporter activity (GO:0005215). KEGG analysis was performed to understand the higher order functional information of biological system^17^. Based on the analysis, a total of 34,140 sequences were annotated with five categories on 232 KEGG pathways (Supplement 1c).

### Analysis of differentially expressed genes (DEGs)

#### Identification of DEGs

A total of 584 annotated transcripts showed significant differential expression in at least one comparison (HT vs NT, HT vs LT and LT vs NT) (adjusted q-value < 0.05). Among them, 362 (223 up-regulated and 139 down-regulated) differentially expressed genes (DEGs) were identified in the HT vs NT group, and 421 (198 up-regulated and 223 down-regulated) DEGs and 113 (74 up-regulated and 39 down-regulated) DEGs were found in the HT vs LT groups and the LT vs NT groups, respectively (Supplement 2). The heat map presents the differently expressed transcripts and shows that LT and NT clustered in one group, indicating that cold stress causes fewer changes than heat stress in black rockfish (Supplement 3).

**Figure 1:**
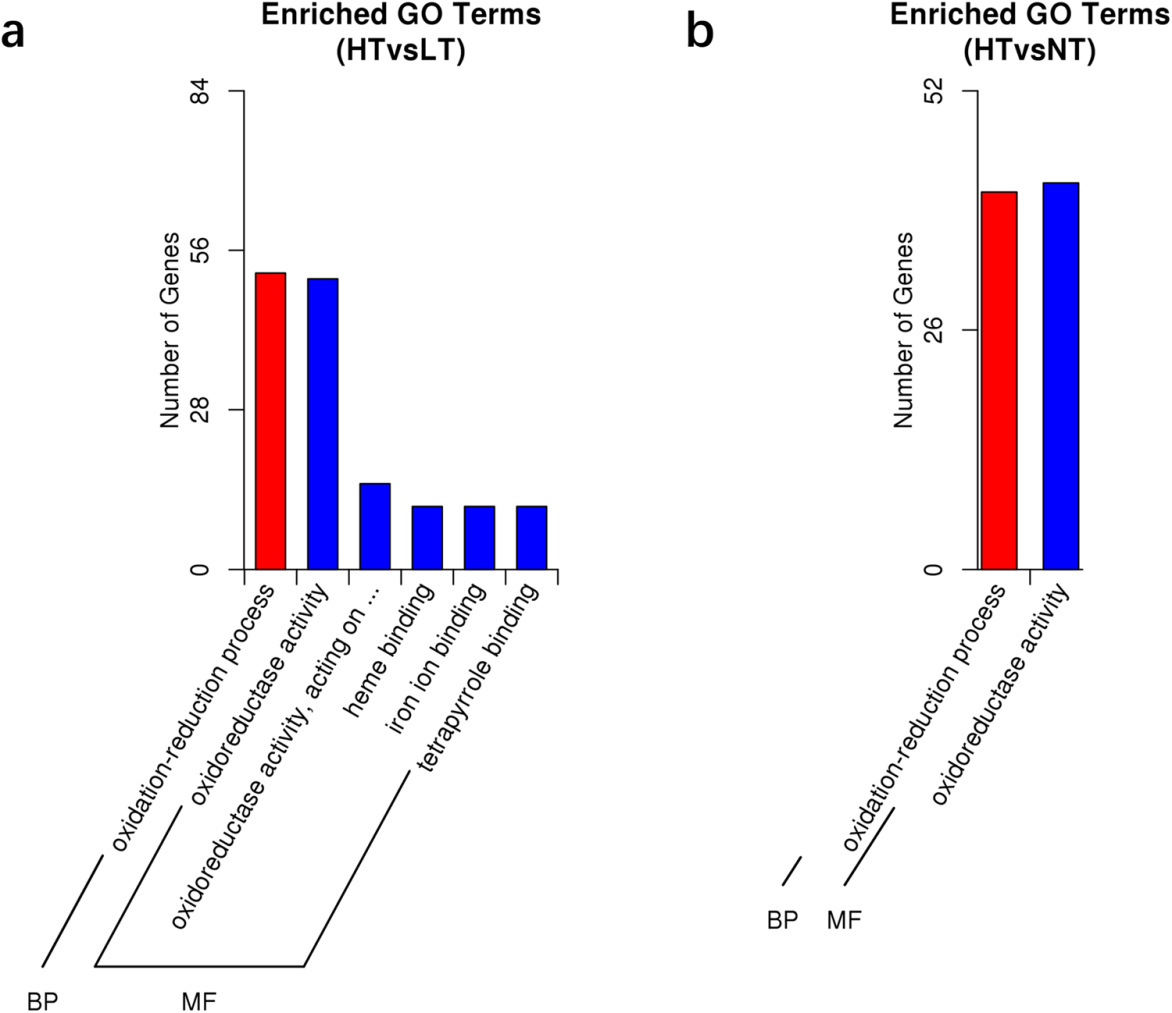
GO enrichment analysis of the differentially expressed genes of the (**a**) HT vs LT group and (**b**) HT vs NT group in the liver of black rockfish. The x-axis shows the specific GO terms. The y-axis shows the number of DEGs for each term.

GO enrichment analysis^18^ was performed on the 584 DEGs. In the HT vs LT group, a total of 151 DEGs has represented significantly enriched GO classifications, including 52 DEGs assigned oxidation-reduction process (BP, GO:0055114), 51 DEGs assigned oxidoreductase activity (MF, GO:0016491), 15 DEGs in oxidoreductase activity, acting on paired donors, with incorporation or reduction of molecular oxygen (MF, GO:0016705), 11 DEGs assigned heme binding (MF, GO:0020037), 11 DEGs assigned iron ion binding (MF, GO:0005506) and 11 DEGs in tetrapyrrole binding (MF, GO:0046906) (Fig. 1a). In HT vs NT group, only 83 DEGs represented an enriched GO classification, including 41 DEGs in oxidation-reduction process (BP, GO:0055114) and 42 DEGs assigned oxidoreductase activity (MF, GO:0016491) (Fig. 1b). There was no DEG enrichment observed for the GO classification of the LT vs NT group.

DEGs were mapped to several specific pathways by the KEGG pathway analysis, which included 194, 189 and 75 KEGG pathways in the HT vs LT group, HT vs NT group and LT vs NT group, respectively. Here, we present 26 significantly enriched pathways (q-value < 0.05); of these, the HT vs LT group mainly contains 11 pathways, including influenza A (KO: 05164), legionellosis (KO: 05134) and estrogen signaling pathway (KO: 04915); the HT vs LT group mainly contains 14 pathways including antigen processing and presentation (KO:04612) and NOD-like receptor signaling pathway (KO: 04621), and the LT vs NT group contains the PPAR signaling pathway (KO: 03320) (Supplement 4**)**.

Based on the KEGG pathway analysis^17,19,20^ and manual literature searches, a total of 245 candidate genes associated with stress responses and adaptation were identified in the 584 annotated DEGs; these candidate genes were filtered and classified into 8 functional categories including protein folding, metabolism, immune response, signal transduction, molecule transport, membrane, and cell proliferation/apoptosis (Supplement 5). A total of 100 genes with a |log2(fold change)| > 2 were selected from the 245 candidate genes and are listed in Table 3. The imputed putative functions of these genes are covered in the Discussion.

**Table 3:** Enriched DEGs potentially associated with temperature stress adaptation in liver of black rockfish.

Function classifications	DEG gene id	Gene name	Log2(fold change)		
			HT vs LT	HT vs NT	LT vs NT
protein folding					
	c192313_g1	dnaJ homolog subfamily A member 4 (DnaJA4)	**3.1309**	**4.3802**	1.1948
	c24203_g1	dnaJ homolog subfamily B member 1-like (DnaJb1)	**8.2766**	**8.6407**	0.30959
	c211750_g1	dnaJ homolog subfamily B member 9-like (DnaJb9)	2.2192	1.8757	−0.39802
	c109290_g4	dnaJ homolog subfamily C member 3-like (DnaJC3)	1.9669	2.2426	0.22118
	c98113_g1	E3 ubiquitin-protein ligase (HERC6)	**−2.6489**	**−2.0253**	0.56912
	c104467_g1	E3 ubiquitin-protein ligase (RNF130)	**3.6561**	**3.804**	0.093412
	c98469_g1	E3 ubiquitin-protein ligase (RNF139)	**5.3642**	**4.5749**	−0.84377
	c107918_g5	E3 ubiquitin-protein ligase (RNF19B)	**5.2773**	**5.9936**	0.66176
	c107608_g1	E3 ubiquitin-protein ligase (TRIM21)	**−3.9276**	**−3.8645**	0.008534
	c130781_g1	E3 ubiquitin-protein ligase KEG-like (KEG)	**5.3772**	**5.7753**	0.34358
	c94103_g1	E3 ubiquitin-protein ligase isoform X1 (NEURL3)	**7.4928**	**6.7601**	−0.78723
	c104218_g1	E3 ubiquitin-protein ligase (HERC4)	**2.5982**	**2.7976**	0.14494
	c109889_g6	G2/M phase-specific E3 ubiquitin-protein ligase (G2E3)	**3.7338**	**6.784**	**2.9957**
	c109681_g15	heat shock 70 kDa protein 4 L (hsp70)	**7.8831**	**8.4998**	0.56222
	c192182_g1	heat shock cognate 71 kDa (hsc71)	1.5499	3.0304	1.426
	c91106_g1	heat shock cognate 70-2 (hsc70)	**2.0097**	**3.4055**	1.3413
	c98553_g1	heat shock protein 30-like (hsp30)	**9.0226**	**9.4412**	0.36414
	c95628_g1	heat shock protein 60 (hsp60)	**2.7473**	**3.7056**	0.90383
	c105798_g1	heat shock protein 70a (hsp70a)	**5.2478**	**7.5167**	**2.2143**
	c107809_g2	heat shock protein 90 alpha (hsp90a)	**7.4007**	**8.6863**	1.2311
	c84785_g1	hsp90 co-chaperone (cdc37)	**2.1511**	**2.4243**	0.21862
	c109619_g1	myelin-oligodendrocyte glycoprotein (MOG)	**4.5874**	**5.2661**	0.62421
	c109431_g2	nascent polypeptide-associated complex subunit alpha (NACA)	**3.2548**	**3.6132**	0.30396
	c106316_g1	nuclear factor erythroid 2-related factor 1-like isoform X2(Nrf2)	**5.3635**	**6.5145**	1.0965
	c71977_g1	peptidyl-prolyl cis-trans isomerase (PIN1)	**2.2325**	**2.8578**	0.57078
	c104420_g1	sequestosome-1 (Sqstm1)	**5.1945**	**5.4886**	0.23954
	c88305_g1	serpin H1 isoform X2 (LOC100707521)	**6.1501**	**5.6738**	−0.53089
	c93266_g2	stress-induced-phosphoprotein 1 (STIP1)	**3.4744**	**3.9697**	0.44079
	c56625_g1	T-complex protein 1 subunit delta	1.2283	2.2239	0.94117
	c102022_g2	T-complex protein 1 subunit zeta-like isoform X1	1.0632	2.1246	1.0068
	c60793_g1	TRAF-interacting protein with forkhead-associated domain (tifa)	**4.1332**	**2.5713**	−1.6164
	c107546_g1	UDP-glucuronosyltransferase 2A2-like isoform X1 (LOC100700159)	−1.8853	−2.3847	−0.55389
	c86299_g1	zinc finger and BTB domain-containing protein 11 (ZBTB11)	−1.7791	0.66447	2.389
metabolism					
	c69856_g1	activator of 90 kDa heat shock protein ATPase homolog	**4.6021**	**5.2412**	0.58459
	c99079_g1	probable aminopeptidase (NPEPL1)	**3.2307**	**4.4227**	1.1375
	c95928_g1	1,25-dihydroxyvitamin D(3) 24-hydroxylase	−3.3182	−1.6122	1.6515
	c101804_g1	15-hydroxyprostaglandin dehydrogenase (NAD)	−2.745	−1.9495	0.74095
	c39965_g1	Apolipoprotein A-IV (Apoa4)	−3.2673	−1.7264	1.4864
	c153134_g1	arginase-2 (arg2)	**6.378**	**4.4396**	−1.9929
	c107215_g2	ATP-dependent RNA helicase DDX18 (ddx18)	**2.5993**	**3.1471**	0.4933
	c108383_g13	cytochrome P450 1A1 (CYP1A1)	**−2.7936**	**−2.1299**	0.60925
	c108374_g2	cytochrome P450 2J6 (cyp2j6)	**−2.272**	**−2.2942**	−0.0767
	c99861_g1	cytochrome P450 4V2 (cyp4v2)	−2.293	−1.7114	0.52714
	c90957_g1	DEXH (Asp-Glu-X-His) box polypeptide 58 (dhx58)	−1.5534	−2.1522	−0.6533
	c105583_g1	egl nine homolog 3	**6.0758**	**5.3066**	−0.82367
	c194139_g1	glutathione peroxidase 1 (GPX1)	1.5493	2.2102	0.6064
	c88113_g1	glyceraldehyde 3-phosphate dehydrogenase isoform 2 (gapdh2)	**2.3911**	**2.1934**	−0.2522
	c103259_g1	Glycine dehydrogenase (gldc)	**2.1981**	**2.4335**	0.18097
	c102433_g1	insulin-induced gene 1 protein (insig1)	3.4222	1.8503	−1.6265
	c96407_g1	L-lactate dehydrogenase A chain (LDHA)	**3.1239**	**4.034**	0.85553
	c102626_g1	Methionine sulfoxide reductase B3 (MsrB3)	**6.0752**	**6.2626**	0.13293
	c81886_g1	NADH dehydrogenase subunit 3 (MT-ND3)	**−3.0729**	**−2.273**	0.7454
	c101610_g1	NADH dehydrogenase subunit 4 (mitochondrion) (MT-ND4)	−2.6648	−1.8552	0.75517
	c90918_g1	NADH dehydrogenase subunit 5 (mitochondrion) (MT-ND5)	−2.4665	−1.69	0.72208
	c2262_g1	procollagen-lysine, 2-oxoglutarate 5-dioxygenase 3 (plod3)	**2.9351**	**3.7245**	0.73487
	c109198_g2	prolyl 4-hydroxylase, alpha polypeptide II (p4ha2)	**3.1278**	**2.2908**	−0.89158
	c110121_g1	ReO_6	−3.0723	−0.94033	2.0775
	c94607_g1	Retinoic acid receptor responder protein 3(RARRES3)	**−2.7123**	**−3.0623**	−0.40448
	c44652_g1	suppressor of G2 allele of SKP1 homolog isoform X2 (Sugt1)	**4.1201**	**3.9336**	−0.24096
	c101986_g1	tetraspanin-8-like (LOC107862373)	**2.3998**	**2.816**	0.36163
	c101378_g1	thioredoxin reductase 1 (TXNRD1)	**2.5668**	**2.7604**	0.13908
	c83946_g1	torsin-4A-B-like (TOR4A)	1.7851	2.5266	0.687
	c106502_g1	Tumor necrosis factor alpha-induced protein 3(TNFAIP3)	2.641	1.9235	−0.772
	c31528_g1	UPF0444 transmembrane protein	**5.2695**	**4.9729**	−0.35107
	c110228_g2	uridine phosphorylase 1 (UPP1)	**4.3892**	**5.1122**	0.66842
signal transduction					
	c60687_g1	ADP-ribosylation factor-like protein 5C (arl5c)	**3.7238**	**3.0499**	−0.72833
	c106983_g4	AN1-type zinc finger protein 2A	**5.6427**	**6.2023**	0.50503
	c85819_g1	calcipressin-1 isoform X3	**3.9171**	**4.1328**	0.1612
	c99118_g1	dual specificity protein phosphatase 1 (DSPTP1)	**2.1298**	**2.4919**	0.30755
	c107805_g2	dyslexia-associated protein KIAA0319 homolog isoform X2	**7.1164**	**8.8624**	1.6915
	c91626_g1	Early growth response protein 1 (EGR1)	1.3741	**3.9589**	**2.5303**
	c109432_g4	hypothetical protein EH28_02850	**6.0605**	**5.8659**	−0.24912
	c104988_g3	MAP kinase-interacting serine/threonine-protein kinase 2 (MNK2)	**2.3791**	**2.636**	0.20236
	c61170_g1	protein phosphatase 1 regulatory subunit 15A-like (LOC104921782)	**4.264**	**5.2205**	0.90201
	c59834_g1	proto-oncogene (c-Fos)	**4.848**	**5.2862**	0.38372
	c101082_g1	serine/threonine-protein kinase isoform X1 (Sgk1)	**3.2381**	**4.0319**	0.73931
	c106934_g1	transcription factor AP-1 (c-Jun)	**4.5427**	**3.2983**	−1.2988
cell proliferation/apoptosis					
	c93860_g1	chromobox protein homolog 8-like (LOC103373459)	**4.7282**	**5.102**	0.31936
	c56129_g1	BAG family molecular chaperone regulator 3 isoform X1(bag3)	**5.899**	**6.5849**	0.63138
	c91924_g2	CCAAT/enhancer-binding protein delta (CEBPD)	1.2304	2.0995	0.81458
	c106620_g1	cyclin-G2(CCNG2)	**4.1148**	**3.9667**	−0.20261
	c79103_g1	DNA damage-inducible transcript 4 protein (DDIT4)	**5.1599**	**5.8048**	0.59042
	c108412_g1	low-density lipoprotein receptor-related protein 5 (LRP5)	**2.3845**	**2.2381**	−0.20083
	c78608_g1	Signal transducer and activator of transcription 1 (Stat1)	−1.9435	−2.0883	−0.19925
	c100476_g1	tubulin-folding cofactor B-like isoform X1 (LOC105780647)	1.5826	2.1021	0.465
membrane					
	c88063_g1	annexin A2(anxa2)	**4.09**	**4.378**	0.23344
	c106357_g1	Phospholipid scramblase 1 (PLSCR1)	**5.6148**	**6.0483**	0.37891
	c108207_g6	syndecan-4-like (LOC109522306)	**2.6775**	**2.8624**	0.13041
	c100526_g2	zinc finger protein (ZPR1)	**2.133**	**2.4767**	0.28918
	c89547_g1	zinc finger protein 36 (zfp36)	2.0513	1.3348	−0.77103
immune response					
	c111546_g1	C-X-C motif chemokine 11 (CXCL11)	**2.6117**	**3.2039**	0.53763
	c55009_g3	L-rhamnose-binding lectin CSL2-like (LOC106605779)	2.4509	1.5288	−0.97656
	c110865_g1	von Willebrand factor A domain-containing protein 7-like (LOC107083484)	2.6218	0.70835	−1.9679
	c98046_g1	Receptor-transporting protein 3 (RTP3)	−1.5465	−2.7174	−1.2253
	c103655_g1	interleukin-1 beta (IL-1β)	**7.3161**	**5.9328**	−1.4378
molecule transport	c105348_g1	bile salt export pump (ABCB11)	−2.3561	−1.1837	1.1179
	c108297_g1	ubiquitin-protein ligase E3C (UBE3C)	**2.9169**	**2.8331**	−0.13827
others	c107603_g2	serum amyloid P-component-like (LOC109196457)	**−4.6396**	−0.9656	**3.6195**
	c82313_g1	transcription factor jun-B-like (LOC104959396)	1.0688	2.2742	1.1508
	c89416_g1	uncharater gene	**−2.4518**	**−4.4257**	**−2.0284**

Input gene names in bold text shows an intersectional gene in both 2 groups or among 3 groups.

#### Comparative expression analysis in HT vs LT group, HT vs NT group and LT vs NT group

**Figure 2:**
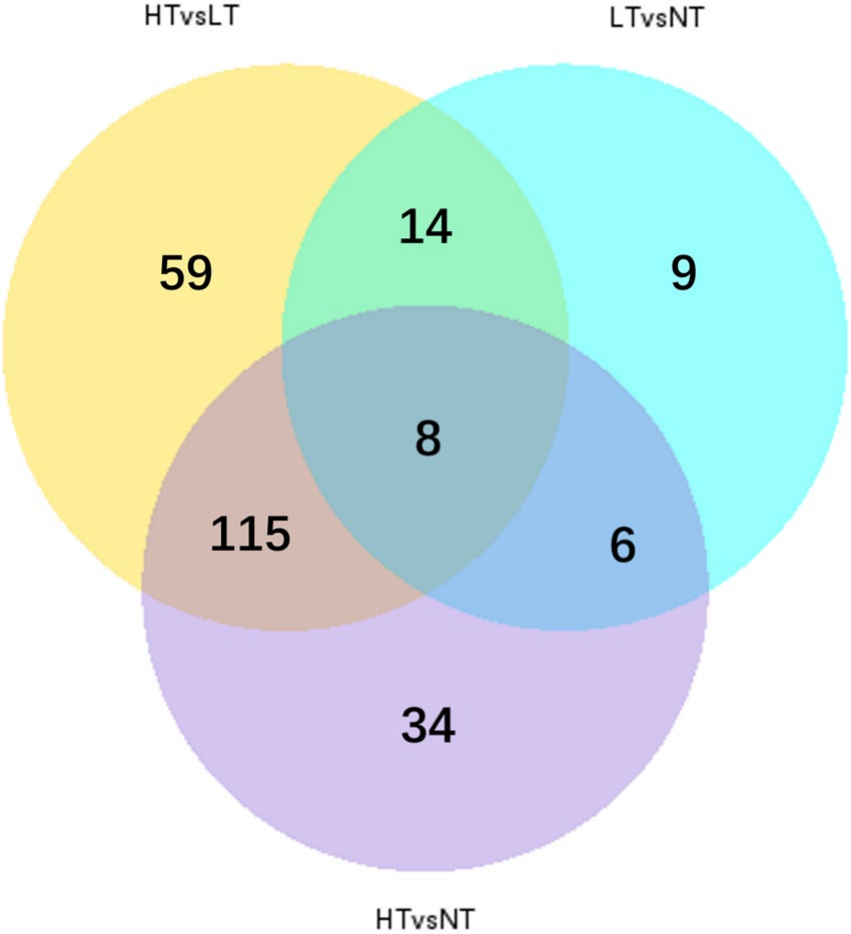
Venn diagram of the filtered DEGs of the HT vs LT group, HT vs NT group and LT vs NT group in the liver of black rockfish.

Based on the 245 DEGs mentioned above, 196, 163 and 37 annotated DEGs were obtained from the HT vs LT group, HT vs NT group and LT vs NT group, respectively (Supplement 5) (Fig. 2).

Among the 196 DEGs from the HT vs LT group, 109 up-regulated and 87 down-regulated DEGs were significantly enriched with 6 GO terms (q-value < 0.05) **(**Fig. 1a**)**. Further analysis shows that 11 DEGs including *1,25-dihydroxy vitamin D(3) 24-hydroxylase cyp1a1, cyp3a4, cyp4v2, dimethylaniline monooxygenase [N-oxide-forming] 5-like, dimethylaniline monooxygenase [N-oxide-forming] 2-like, hmox1, pld, ReO_6, sulfide quinone oxidoreductase, cyp2j6* involved in 3 oxidation-related GO terms (oxidation-reduction process, oxidoreductase activity and oxidoreductase activity, acting on paired donors, with incorporation or reduction of molecular oxygen), and 7 DEGs, *1,25-dihydroxyvitamin D(3) 24-hydroxylase, cyp2j6, cyp1a1, dnajc3, cyp4v2, cox1, cyp3a4* involved in 3 molecule binding-related GO terms (heme binding, iron ion binding and tetrapyrrole binding). Notably, 5 DEGs, 1,25-dihydroxy vitamin D(3) 24-hydroxylase, *cyp1a1, cyp4a2, cyp3a4* and *cyp2j6* were observed in all 6 GO terms, as well as in linoleic acid metabolism pathway (ko00591) (Supplement 4). A total of 163 DEGs were annotated in the HT vs NT group with 111 up-regulated and 52 down-regulated DEGs, which were mainly enriched in 2 GO terms (q-value < 0.1), oxidation-reduction process (GO:0055114; 19 up and 19 down DEGs) in BP and oxidoreductase activity (GO:0016491;20 up and 19 down DEGs) in MT, with 34 shared common genes. This evidence indicates that acute thermal stress on black rockfish may cause an oxidation-reduction change primarily related to the heat damage in the liver^7,21^. In addition, 8 DEGs were filtered form the HT vs LT vs NT comparisons (Fig. 2), 1 gene related to signal transduction (*early growth response protein 1*), 1 gene related to molecule transport (*bile salt export pump, abcb11*), 1 gene related to protein folding (*hsp70a*), 1 gene related to immune responses (*rtp3*), 2 genes involved in metabolism (*1,25-dihydroxyvitamin d(3) 24-hydroxylase, apoa4*), and 2 genes involved in other functions (unchartered gene, *transcription factor jun-b-like*).

**Figure 3:**
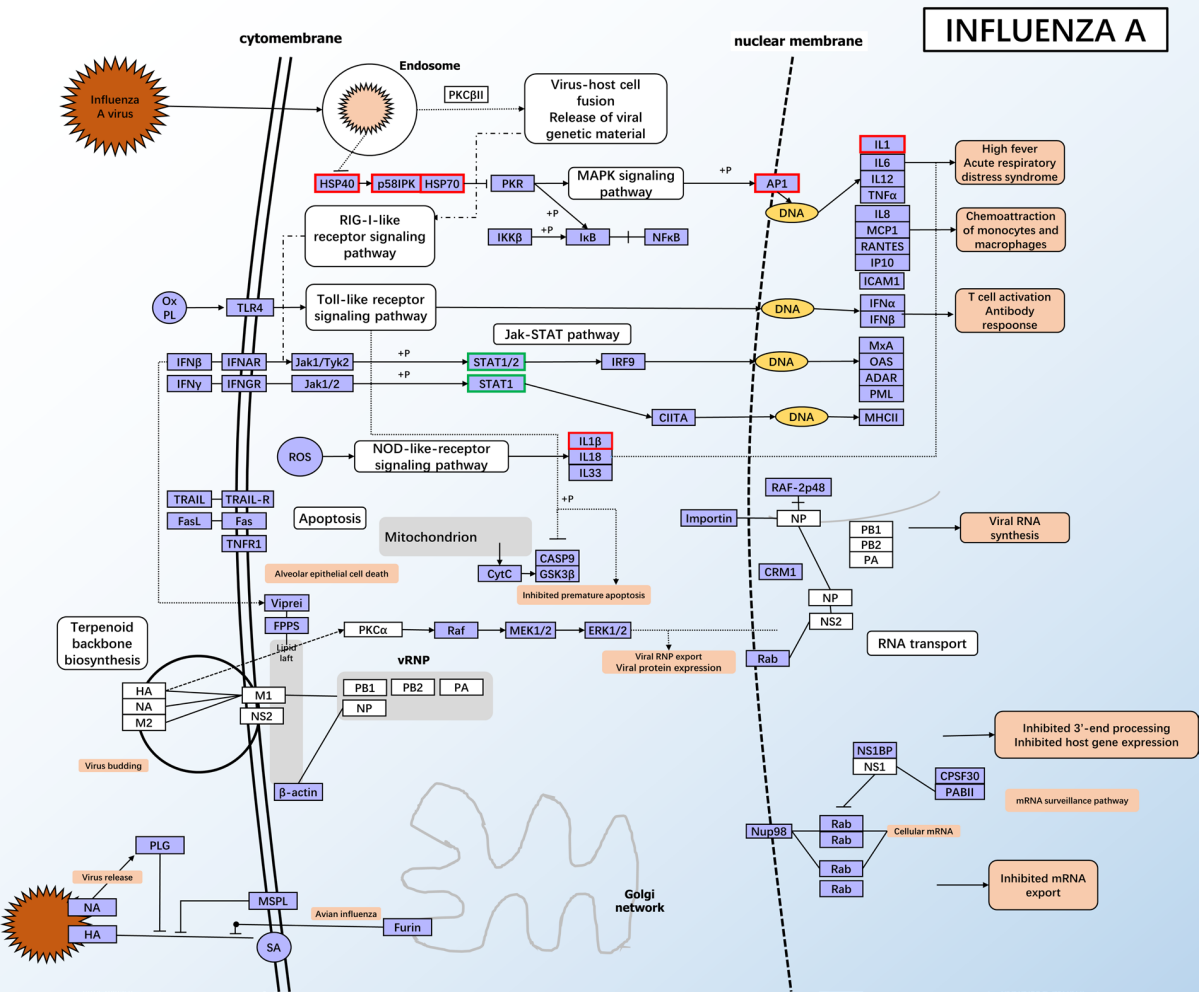
KEGG pathway of the significantly enriched influenza A pathway. Red and green outlines represent up-regulated DEGs and down-regulated DEGs, respectively^17,19,20^.

**Figure 4:**
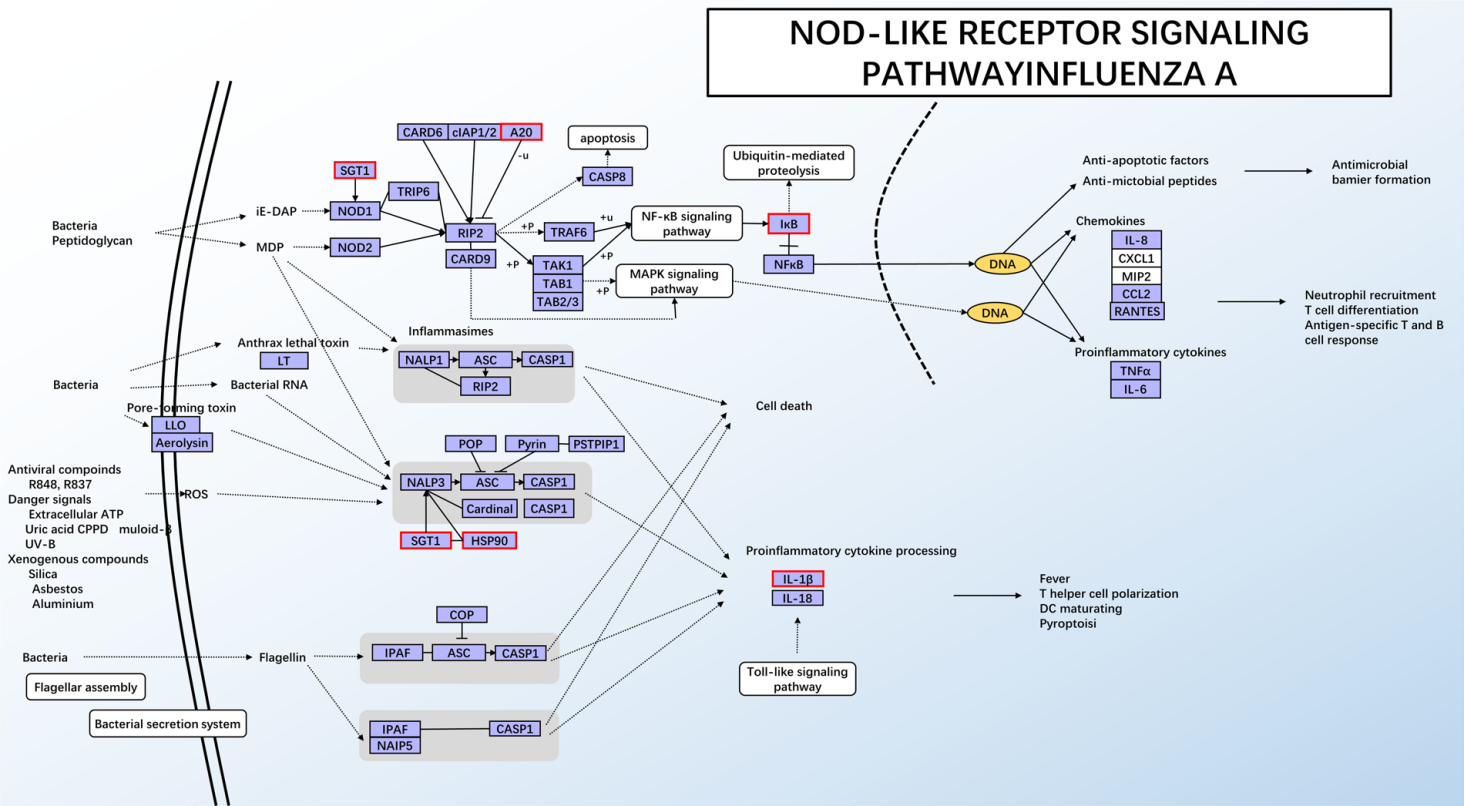
KEGG pathway of the significantly enriched NOD-like receptor signaling pathway. Red and green outline represent up-regulated DEGs and down-regulated DEGs, respectively^17,19,20^.

**Figure 5:**
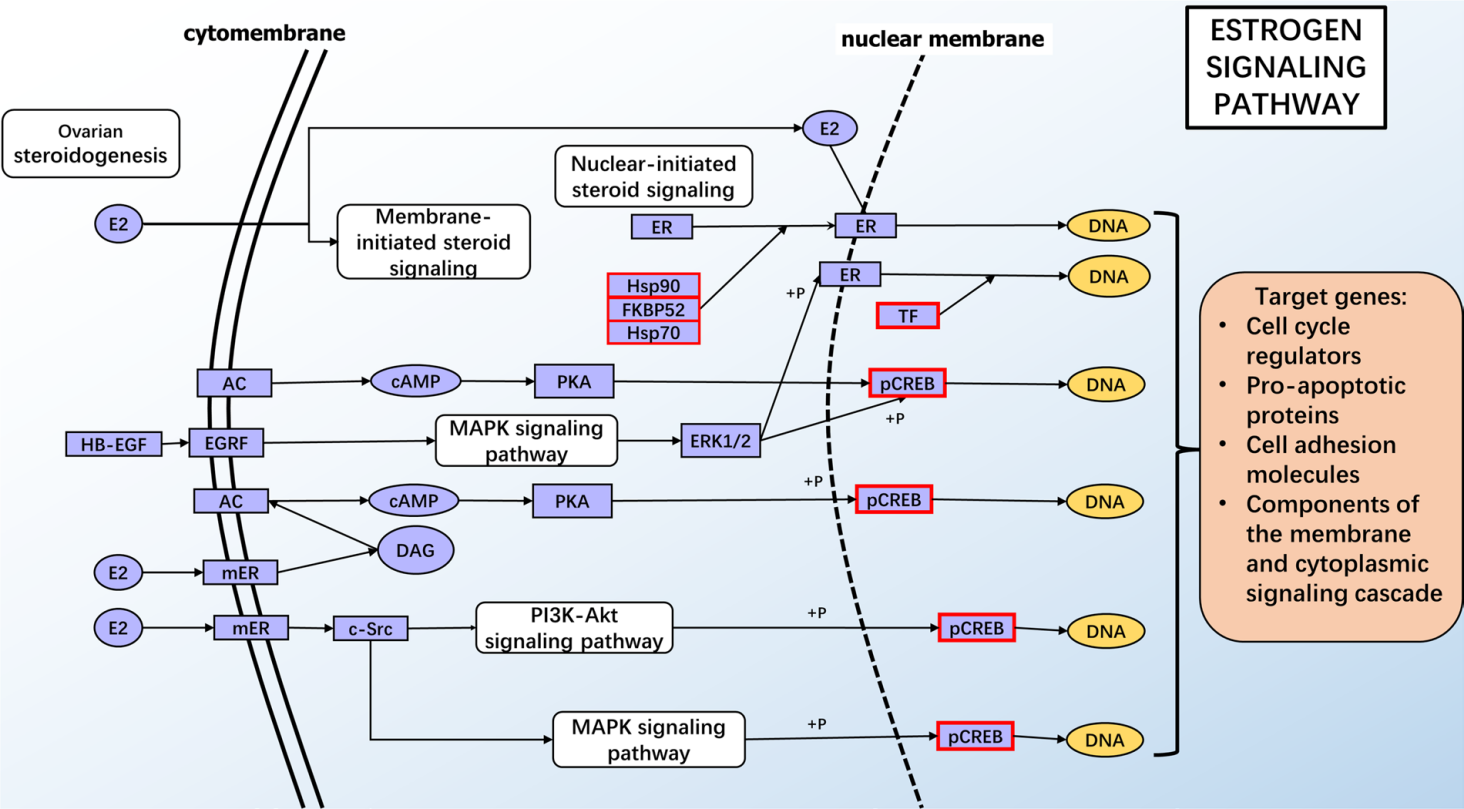
KEGG pathway of the significantly enriched estrogen signaling pathway. Red and green outlines represent up-regulated DEGs and down-regulated DEGs, respectively^17,19,20^.

For the intersection analysis among the different groups comparisons, a total of 123 DEGs were shared between the HT vs LT and the HT vs NT groups, which presented the maximum numbers of shared DEGs among all the intersections (Fig. 2). Most notably, 9 DEGs- enriched KEGG pathways (Table 4) were shared between the HT vs LT and the HT vs NT groups (Table 4). GO enrichment analysis suggested 26 genes were enriched in the oxidation-reduction process term (13 up and 13 down DEGs) in BP and 26 were enriched in the oxidoreductase activity term (13 up and 13 down DEGs) in MT, including 24 DEGs sharing both GO terms (Supplement 2). Nine KEGG pathways were selected from the intersection of the HT vs LT group and the HT vs NT group, which were enriched in 11 and 14 KEGG pathways, respectively (q-value < 0.05) (Supplement 4). Among these 9 KEGG pathways, four pathways, influenza A **(**Fig. 3**)**, legionellosis, inflammatory bowel disease (IBD) and measles were related to immune and infectious diseases. Four pathways, NOD-like receptor signaling pathway **(**Fig. 4**)**, osteoclast differentiation, plant-pathogen interaction IBD and antigen processing and presentation, were related to organismal systems especially the immune system, and the estrogen signaling pathway **(**Fig. 5**)** was related to the endocrine system.

**Table 4:** 9 KEGG pathways enriched in both the HT vs LT group and the HT vs NT group.

Term&ID	Background/Input (HTvsLT, HTvsNT) gene number	HTvsLT			HTvsNT		
		q-Value	Input gene name		q-Value	Input gene name	
Influenza A	424/14,19	0.004754	***hsc70***	***stat1***	7.27E-06	***hsc70***	***stat1***
ko05164			***hsp70a***	*alias p85α*		***hsp70a***	*cyt c-b*
			***dnajc3***	*ap-1*		***dnajc3***	*mda5*
			***c-jun***	*ivns1abp*		***c-jun***	*β actin*
			***il-1β***			***il-1β***	*ikkalpha2*
			***ikkalpha***			***ikkalpha***	*viperin*
			***pkr***			***pkr***	*jun*
			***dnajb1-like***			***dnajb1-like***	*furin-1*
			***dnajb1***			***dnajb1***	*cyt c*
			***hsc71***			***hsc71***	
Legionellosis	182/9,11	0.004754	***hsc70***	***ikkalpha***	0.00018	***hsc70***	***ikkalpha***
ko05134			***hsp70a***	***hsc71***		***hsp70a***	***hsc71***
			***hsp60***	*tlr5*		***hsp60***	*cyt c-b*
			***rab1a***	*nf-kappab*		***rab1a***	*cdc48*
			***il-1β***			***il-1β***	*nf-kappab1*
							*cyt c*
Estrogen signaling pathway	406/12,12	0.01639	***hsc70***	***pin1***	0.014251	***hsc70***	***pin1***
ko04915			***hsp90b(2)***	***hsc71***		***hsp90b(2)***	***hsc71***
			***hsp70a***	*creb2*		***hsp70a***	*hsp90b1*
			***c-jun***	*alias p85α*		***c-jun***	*hsp 90b*
			***hsp90***	*ap-1*		***hsp90***	*creb2*
			***hsp90a***	*shc2*		***hsp90a***	*jun*
NOD-like receptor signaling pathway	150/7,9	0.018144	***hsp90a***	*il-1β*	0.001062	***hsp90a***	*hsp90b1*
ko04621			***hsp90b(2)***	*tnfaip3*		***hsp90b(2)***	*hsp 90b*
			***hsp90***			***hsp90***	*nf-kappab1*
			***ikkalpha***			***ikkalpha***	*il-1β*
			***sugt1***			***sugt1***	
Osteoclast differentiation	319/10,11	0.021659	***sqstm1***	*nf-kappab*	0.009519	***sqstm1***	*jun*
ko04380			***junb***	*alias p85α*		***junb***	*nf-kappab1*
			***c-jun***	*il-1β*		***c-jun***	*junb-like*
			***ikkalpha***	*ap-1*		***ikkalpha***	*il-1β*
			***sqstm1***			***sqstm1***	*fosl1*
			***stat1***			***stat1***	
Plant-pathogen interaction	85/5,7	0.027236	***tufm***	***hsp90***	0.001062	***tufm***	***hsp90***
ko04626			***hsp90a***			***hsp90a***	*hsp90b1*
			***hsp90b(2)***			***hsp90b(2)***	*hsp 90b*
			***sugt1***			***sugt1***	
Inflammatory bowel disease (IBD)	87/5,5	0.027236	***il-1β***	*tlr5*	0.027087	***il-1β***	*stat3*
ko05321			***stat1***	*ap-1*		***stat1***	*jun*
			***c-jun***			***c-jun***	
Measles	309/9,10	0.038681	***hsc70***	***hsc71***	0.016579	***hsp70a***	***hsc71***
ko05162			***hsp70a***	***stat1***		***il-1β***	***stat1***
			***il-1β***	*alias p85α*		***ikkalpha***	*stat3*
			***ikkalpha***	*tnfaip3*		***pkr(2)***	*mda5*
			***pkr(2)***			***hsc70***	*nf-kappab1*
Antigen processing and presentation	204/7,8	0.045485	***hsc70***	***hspa4***	0.016579	***hsc70***	***hspa4***
ko04612			***hsp90b(2)***	***hsp90a***		***hsp90b(2)***	***hsp90a***
			***hsp70a***	***hsc71***		***hsp70a***	***hsc71***
			***hsp90***			***hsp90***	*hsp 90b*

Input gene names shown in bold text belong to both groups in each KEGG pathway.

There were only 37 DEGs (25 up-regulated and 12 down-regulated) identified in the LT vs NT group, which further indicated cold stress may cause less changes than thermal stress.

### Validation of RNA-Seq results by qRT-PCR

**Figure 6:**
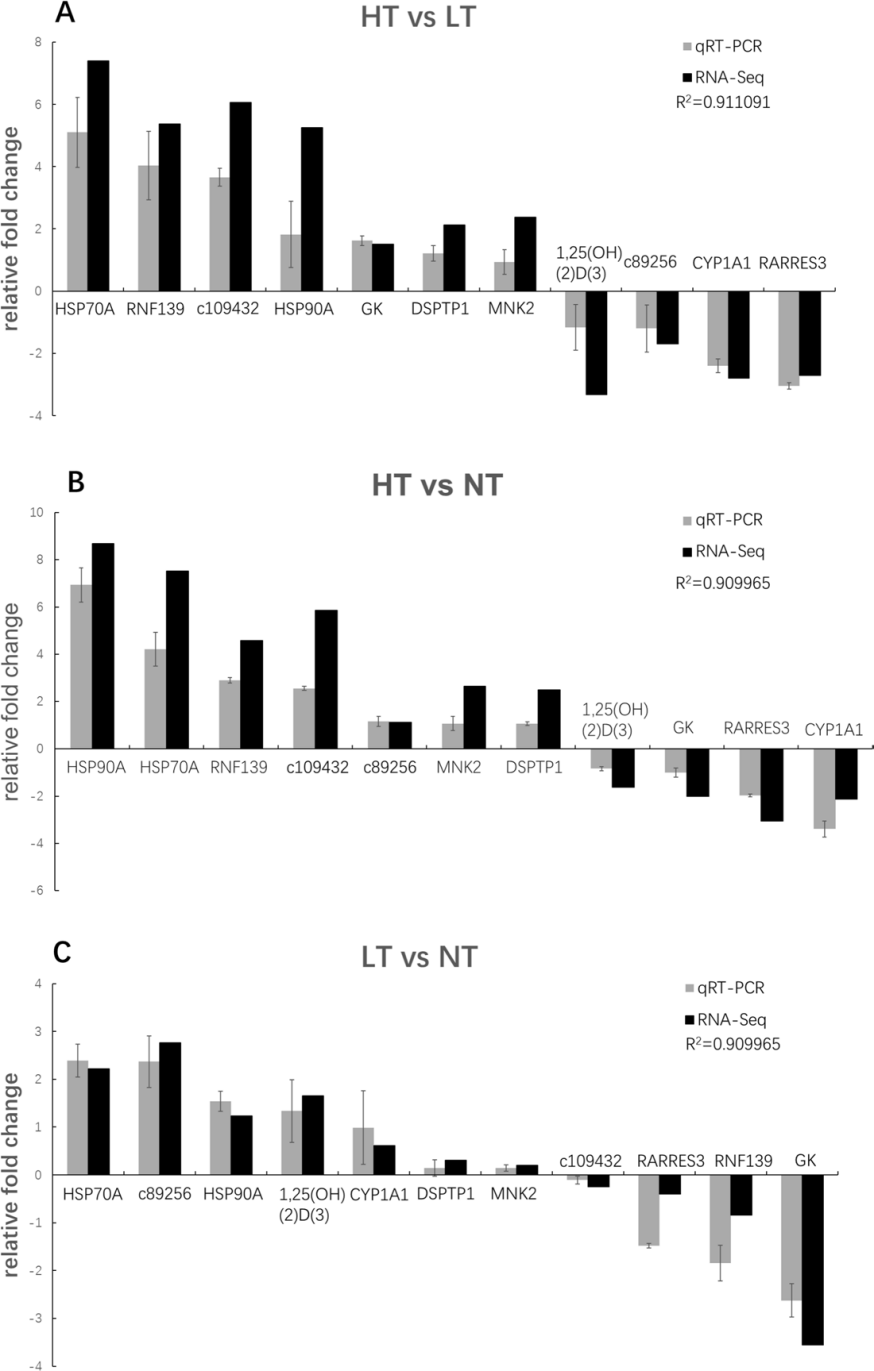
qRT-PCR validation of 11 differentially expressed genes generated from RNA-Seq results from the black rockfish liver. The expression levels of the selected genes were normalized to the 18S gene. (**a**) HT vs LT; (**b**) HT vs NT; (**c**) LT vs NT. Gene abbreviations are: retinoic acid receptor responder protein (*RARRES3*); heat shock protein 70a (*HSP70A*); heat shock protein 90 alpha (*HSP90A*); 1,25-dihydroxyvitamin D(3) 24-hydroxylase (1,25(OH)(2)D(3)); dual specificity protein phosphatase 1 (*DSPTP1*); cytochrome P450 1A1 (*CYP1A1*); E3 ubiquitin-protein ligase (*RNF139*); MAP kinase-interacting serine/threonine-protein kinase 2 (*MNK2*); c109432; glucokinase (*GK*).

To validate the RNA-Seq results, 11 DEGs were randomly selected for qRT-PCR analysis (Supplement 6). The results showed that the qRT-PCR expression trends of the selected genes were significantly correlated with the RNA-Seq results (R^2^: 0.882–0.911). Generally, the RNA-Seq results were confirmed by the qRT-PCR results, implying the reliability and accuracy of the RNA-Seq analysis **(**Fig. 6**)**.

## Discussion

Fish exposed to thermal/cold conditions will show some signs of stress, which results in the depression of immune responses^22^, reproduction^23^, energy metabolism^7^ and growth^2^. In November, the surface temperature of the northwestern Pacific Ocean was unstable, and the temperature difference in one photoperiod varied from 7 °C to 20 °C. Under this environment, the black rockfish may experience serious acute temperature stress, which will cause heat shock, disease, and metabolism and reproduction problems. However, studies investigating the molecular mechanisms under temperature stress in black rockfish are still lacking. As studies have shown that the liver is one of the most important organs for metabolism adjustments in the process of stress adaptations^24^, in the present study, we conducted an RNA-Seq analysis on liver samples to reveal the molecular mechanisms underlying the response to temperature stress in black rockfish.

A total of 250,326 transcripts were generated with 66,596 (30.7%) transcripts yielding the Nr databases match, which greatly enriched the transcriptome data of black rockfish. This study not only identified potentially differentially expressed transcripts under acute thermal/cold conditions but also identified many new annotated gene sequences in black rockfish.

To maintain homeostasis under acute stress, energy supply and immune response pathways are activated, along with the activation of material synthesis, metabolic activity and signal pathways. In this present study, a total 584 annotated transcripts were identified in the black rockfish liver during the three comparisons (HT, LT and NT) in response to temperature stress. These differentially expressed genes were enriched and categorized based on a GO annotation, KEGG enrichment analysis and manual literature search, and several key genes or pathways likely involved in responses/adaptions to temperature stress were highlighted, as discussed below.

### Candidate genes or pathways involved in the heat stress response

In this study, 8 differently expressed genes were identified by the HT vs LT vs NT comparison (Fig. 4**)**: 1 gene related to signal transduction (*early growth response protein 1*), 1 gene related to molecule transport (*bile salt export pump*, *abcb11*), 1 gene related to protein folding (*hsp70a*), 1 gene related to immune responses (*rtp3*), 2 genes involved in metabolism (*1,25-dihydroxyvitamin d(3) 24-hydroxylase, apoa4*), and 2 genes involved in other functions (unchartered gene, *transcription factor jun-b-like*). Therein, HSP70 is a charter stress response gene and has been mentioned along with *Apoa4* in a previous study. Heat shock stress is considered to be a well-known and studied stressor. In a study on grass carp (*Ctenopharyngodon idellus*), HSP70 gene expression was found to be up-regulated in spleens under high temperature stress^22^. Early growth response protein 1 (EGR1) indicates that the DNA methylation status of the promoter under stress plays a crucial role in the consolidation of immobility behavior^25^. Transcription factor jun B is a part of the inducible transcription factor complex AP-1, which is quickly activated during gravity alterations and regulates the formation of primary osteoblasts^26^. Bile salt export pump (ABCB11) functions in bile acid transport and is a susceptive factor in hepatocytes injury^27^, and, similar to the results observed here, it was reduced after heat stress during a previous study on rats^28^. Receptor transporting protein 3 (*RTP3*) was found to be associated with virus infection in Asian seabass^29^.

Among the 9 KEGG pathways enriched in both the HT vs LT group and the HT vs NT group, 4 KEGG pathways (influenza A, legionellosis, lnflammatory bowel disease (IBD) and measles) were related to immune and infectious diseases, and 4 KEGG pathways (NOD-like receptor signaling pathway, osteoclast differentiation, plant-pathogen interaction inflammatory bowel disease (IBD) and antigen processing and presentation) were related to the immune system. Considering that heat stress has a negative effect on the inner immune system^30,31^, it is no wonder that infection-related pathways (e.g., influenza A) and immune system response pathways (e.g., NOD-like receptor signaling pathway) are activated after stress treatments.

Influenza A viruses are the agents for a disease that can lead to high morbidity^32^ (Fig. 3), and legionellosis is a disease caused by *Legionella* cell infection^33^. In a study on mice under chronic heat stress, the inner immune system of mice was affected and infected with the influenza virus^34^. The immune system is reported to be affected by thermal stress^30,31^, so we can infer that (1) the intracellular immune system may be damaged by thermal stress, leading to infection by some pathogens, or (2) intracellular or intercellular compounds (such as protein and deoxyribonucleic acid) may be damage, activating protein folding and degradation progresses^35^. Five DEGs (*hsc70, hsp70a, hsc71, il-1β and ikkalpha*) were enriched in both influenza A and legionellosis pathways, which are closely related to the immune system. Among the 5 DEGs, *hsc70, hsp70a*, and *hsc71* are three typical stress-related genes. In Atlantic salmon (*Salmo salar*) and brook charr (*Salvelinus fontinalis*), a similar response, that hsp70 protein levels increased under both thermal and cold conditions, was observed^36^. Furthermore, triploids of these two species have the same hsp70 level tendency, with a relatively low concentration compared with the diploids^36^. It is interesting that the heat shock cognate 70–2 (*hsc70*) was enriched in 5 KEGG pathways (corrected p-value < 0.05): influenza a (ko05164); legionellosis (ko05134); estrogen signaling pathway (ko04915); measles (ko05162); antigen processing and presentation (ko04612), indicate that *hsc70* plays a comprehensive role in the acute thermal stress response, a result that has been shown in different species^4,37,38^. Similar to the present results, other studies have reported that hsc71 is up-regulated after hypoxia stress^39^. Interleukin 1 beta (IL-1β) is an evolutionarily conserved molecule originally identified in the immune system, and it plays a critical role in the activation of immune cells^40^. Notable, a study by Tort L showed that the fish immune response is activated under acute stress, but is suppressed under chronic stress^41^. In addition, a study on the heat shock responses of rats suggest that IL-1b plays a major role in heat-induced liver damage, and plays an important role in hepatocyte apoptosis in heat-induced liver injury^42^. In this work, the potentially differentially expressed genes with critical roles in immune responses were functionally annotated (Table 3). Interleukin-1 beta (*IL-1β*) showed an up-regulation trend in the HT treatment, similar to the results reported by a study on the Chinese brown frog (*Rana dybowskii*)^40^, a vertebrate, as well as results reported by a study on the skeletal muscles of *Sebastes schlegelii*^43^. In addition, in this study, *ikkalpha* was up-regulated after acute heat stress. However, in some other heat stress studies, the ikkalpha protein was found to be depleted and phosphorylated in male Sprague-Dawley rats^44^ or coprecipitated with Hsp90^45^.

Thermal stress can result in serious stress-associated inflammatory and metabolic changes^46^. The main function of the NOD-like receptor signaling pathway **(**Fig. 4**)** is inflammasome activation^47^, and osteoclast differentiation has been reported to be associated with the immune system^48^. In this study, black rockfish were under acute thermal stress, and pathogen infections may activate the organismal immune system, causing serious pathway activation. It is well known that ERs participate in the transcription complex with a number of chaperones and cofactors, including HSPs^49^. ER-binding HSP90 is accessible for hormone binding; furthermore, hormone binding promotes a receptor isolated from HSP90, converting it into a DNA-binding state^50^. The sugt1 protein has been shown to be a binding partner of heat shock proteins, and has been found to increase after heat stress^51^, and sugt1, along with hsp90, is found to be essential in both mammalian and plant innate immune responses^52^. In the 4 pathways related to the immune system mentioned above, *c-jun* was up-regulated in the osteoclast differentiation and IBD pathways, and a similar result was found in mice skeletal muscles after heat stress^43^. It has been suggested that *c-jun* may participate in signal transcription to induce an early stress-induced immune response^43^.

Four *hsp* genes and 1 *pin1* gene related to protein folding were enriched in the estrogen signaling pathway in the liver of black rockfish after acute thermal stress **(**Fig. 5**)**. A similar *hsp70/90* up-regulating response was observed in rainbow trout^53^, which suggests that *hsp90* is necessary for vitellogenin induction which is the production of the estrogen signaling pathway.

### Metabolism

Temperature changes may influence aspects of metabolism especially in the oxidant reduction process^6^, and responses to stress are energy-costing processes^7^. In our results, some differently expressed genes involved in glycogen synthesis, fatty acid synthesis and oxidant reduction were overexpressed in the liver.

Malate dehydrogenase (*MDH*) is one key enzyme in the conversion of malate and oxaloacetate by the NAD/NADH system. It is well known in kinetic studies that NAD/NADH is the first compinent in the reaction of malate to oxaloacetate^54^. Under thermal and cold stress in *Sebastes schlegelii*, *MDH* and NADH-related genes (*MT-ND3, MT-ND4* and *MT-ND5*) were down-regulated in the HT vs LT group. The same result of heat-stress-induced repression in genes encoding enzymes *(MT-ND1*, *MT-ND2* and *MT-ND6*) was revealed in channel catfish (*Ictalurus punctatu*s)^7^. Cytochrome P450 (*CYP*) is a superfamily containing a series of genes encoding P450 enzymes and are found in all aerobic eukaryotes and other vertebrates^55,56^. In a study on the cytochrome p450 metabolic enzymes in cows under heat stress conditions, the relative abundances of *CYP2C* and *CYP3A* were found to be decreased^57^. A study on *Symbiodinium* under both rapid and gradual thermal stress revealed that up-regulation occurred under gradual heat stress before the maximum temperature was reached, and down-regulation occurred under rapid stress and gradual stress after the maximum temperature was reached^58^.

In a study on *Sebastes schlegelii*, *MDH* and *NADH-*related genes (*MT-ND3, MT-ND4* and *MT-ND5*) were down-regulated under acute thermal stress compared with the cold stress group and the control group, which are both involved in the tricarboxylic acid cycle, a crucial pathway in oxidative metabolism. Importantly, *cyp3a4* and the associated linoleic acid metabolism pathway (ko00591) were significantly changed under thermal stress, which is similar with the results of a study investigating mitochondrial functions following hypoxia^59^. Under hypoxia caused by the thermal environment, the anaerobic metabolism level will rise, while oxidative metabolism will be repressed, which results in the down-regulation of the oxidative metabolism enzyme mentioned above. Similar effects on anaerobic metabolism caused by thermal inducement have been observed in other fish species^7,60^. Lactate dehydrogenase (*ldh*) and cytochrome c (*cyt*) were observed to be increased under acute warm conditions in a study on rainbow trout (*Oncorhynchus mykiss*)^61^, which agrees with the results in LT treatment of the present study, suggesting energy consumption and functional impairment in mitochondria. In addition, some other metabolic-related genes, such as glycine dehydrogenase (*gldc*), insulin-induced gene 1 protein (*insig1*), thioredoxin reductase 1 (*txnrd1*) and apolipoprotein A-IV (*apoa4*), all showed significant changes in this study, especially under thermal stress^62,63^.

### Protein folding

Temperature affects protein synthesis, modification and degradation at the cellular level because proteins are denatured or misfolded and then become cytotoxic by forming aggregates^5,7,64^. With increases in the number of damaged proteins, the regulation of the repair and degradation of denatured proteins subsequently activates to maintain homeostasis in the cell^65^, a process in which some chaperone proteins are involved.

Heat shock proteins (HSPs), also known as stress proteins, are among the molecular chaperones that play a fundamental role in the regulation of normal protein synthesis and produced in all cellular organisms exposed to stress^66^. A study on in blue-green damselfish (*Chromis viridis*) observed that HSP70 and HSP60 were both elevated in response to a temperature of 32 °C^65^. The present study observed enrichment of *HSP70*, *HSP90*, and *HSP40* family and other heat shock protein genes in *Sebastes schlegelii*, with a significant elevation of all these *HSPs* observed under acute heat stress (Table 3). HSP40 plays a role in the regulation of HSP70 activity by interacting with both the HSP70 and J domains^7^. HSP90 is essential in the folding and assembly of cellular proteins and is involved in the regulation of kinetic partitioning among folding, translocation and aggregation in the cell^66^, especially under damages caused by thermal and cold stress conditions.

Protein modification, by folding degradation represents a series of complex pathways involving different molecules. Ubiquitin in cells acts as a covalent modifier of proteins in functionalization and degradation, which is dependent on ubiquitin ligase. E3 ubiquitin proteins are the final enzymes in the ubiquitin-proteasome pathway, regulating protein degradation, cell growth and apoptosis in response to environmental accommodation^67^. In addition, stress-induced phosphoprotein 1 (*stip1*) is also known as an HSP70/HSP90 organizing protein, expressed in the heat shock response^68^.

### Signal transduction

Responses and accommodations to different stresses involve a series of comprehensive and complex pathways. G protein-coupled receptor 155 (GPR155) belongs to the seven-transmembrane domain of the GPCRs superfamily^69^. The ligands for the GPCRs have varied ions, amines, proteins and lipids, which may be caused by stress and accommodation^70^. The CREB (cAMP response element binding) protein is a cellular transcription facto that responds to different physiological signals, including stresses^9^. In this study, some differentially expressed genes potentially involved in signals transduction were found in the HT vs LT group, such as G protein-coupled receptor 155 (GPR155), MAP kinase-interacting serine/threonine-protein kinase 2 (MNK2), methionine tRNA ligase and cyclic-AMP response element-binding protein 2 (CREB). They may have important functions in regulation of signaling to activate responses against harm caused by thermal conditions.

### Immune response

Different from mammals or birds, fish are ectothermic, with immune systems exposed to changes in the external temperature^71^. In addition, teleost have a complete vertebrate immune system similar to that of mammals^72^. Previous studies have focused on immune responses within different temperatures ranges in different fish species. Complement C3 protein gene expression increased in the orange spotted grouper (*Epinephelus coioides*) liver under temperature stress, and C3 may play a critical role in immune mechanisms^73^. Some other genes were found to be potentially involved in the *S.schlegelii* heat stress response, such as the C-X-C motif chemokine 11 (*CXCL*), which is an interferon-induced inflammatory chemokine expressed by leukocytes, fibroblasts and endothelial cells^74^, latexin (Lxn) and complement C3. Further immune response mechanisms will studies in more detail in the future.

In conclusion, the results of this study demonstrate that the the acute thermal conditions and stress significantly affect black rockfish (*S. schlegelii*). A total of 584 DEGs were obtained in response to acute thermal (27 °C) and cold (5 °C) stress exposure, such as *hsps, mdh, cyp2c*. These stress-regulated genes are associated with metabolism, protein folding, immune response, cell proliferation/apoptosis, membrane, molecule transport, regulation of transcription and others categories, which enables the understanding of molecular mechanism in response to temperature stress for aquatic species.

## Materials and Methods

### Ethics statement

All procedures involved in handling and treatment of fish during this study were approved by Animal Research and Ethics Committees of Ocean University of China prior to the initiation of the study. The field studies did not involve endangered or protected species. All experiments were performed in accordance with relevant guidelines and regulations.

### Animals

40 male adults of black rockfish cultured by cages were obtained in November from northern Yellow Sea, Shandong province, China. The natural seawater temperature was 16 °C (±0.5 °C). Following capture, fish were acclimatized at a density of 10 individuals per tank (diameter 1 m, height 1.5 m) under laboratory conditions for two days without feeding. Water temperature, dissolved oxygen and salinity were maintained at 16 °C (±0.7 °C), 7.22 mg/L (±0.59 mg/L) and 30 ppm, respectively.

### Temperature challenge and fish sampling

After acclimation, a total of 30 fishes were randomly divided into 3 groups: low temperature group (LT, n = 10), control group (natural temperature, NT, n = 10) and high temperature group (HT, n = 10). The temperatures of the above three groups were set at 5 °C (±0.5 °C), 16 °C (±0.5 °C) and 27 °C (±0.5 °C), respectively. Three water tanks were filled by fresh seawater which was heated using heating rod or cooled down by refrigerator before treating.

Fish were transferred to the three water tanks directly by groups. After 12 h treating, 10 individuals per tank were sampled under 200 mg /L tricaine methanesulfonate (MS-222) anesthesia. Liver samples were collected from all individuals in each treatment, which were frozen immediately by liquid nitrogen and stored at −80 °C for RNA extraction.

### RNA extraction, library construction and transcriptome sequencing

To reduce the variation among individuals, 3 liver tissue samples/ treatment were mixed for RNA extraction. Total RNA was extracted from freshly thawed liver samples using TRIzol^®^ reagent (Invitrogen, USA) and treated with TURBO DNA-free ™ kit (Invitrogen) to remove genomic DNA. The concentration and quality of the total RNA were assessed by Agilent 2100 Bioanalyzer system (Agilent Technologies, USA). Further, equal volume of RNA from 3 mixed samples/ treatment were pooled together in order to mask the difference among repetitions. 6 sequencing libraries totally were generated using NEBNext® Ultra™ RNA Library Prep Kit for Illumina® (NEB, USA) following manufacturer’s instructions and index codes were added to attribute sequences to each sample. Samples were sequenced on an Illumina Hiseq. 4000 platform and 150 bp paired-end reads were generated. Raw sequences were deposited in the Short Read Archive of the National Center for Biotechnology Information (NCBI) with accession numbers of SRR4409372 (NT), SRR4409389 (LT) and SRR4409390 (HT).

### Quality control and *De novo* assembly of sequencing reads

Initially, reads with adapter, reads containing more than 0.1% poly-N and low quality reads were trimmed to generate high quality clean data. Then *de novo* assembly was performed on liver clean reads using the Trinity assembly software suite^75^. Trinity’s assembly pipeline consists of three consecutive modules: Inchworm, Chrysalis, and Butterfly. All overlapping k-mers (k-mer = 25) were extracted from clean reads. Inchworm then examined each unique k-mer and generated transcript contigs using a greedy extension based on (k-1)-mer overlaps. Chrysalis clusters related Inchworm contigs into components, which were encoded by building a de Bruijn graph for each cluster. This clusters together regions that have likely originated from alternatively spliced transcripts or closely related gene families. Finally the Butterfly module processed the individual graphs in parallel, generating final transcripts^76^.

### Annotations of transcripts and pathways

Transcripts (both contigs and singletons) were annotated by performing BLASTx searches^77^ using NCBI non-redundant (Nr), NCBI nucleotide sequences (Nt) and Swiss-Prot databases with a cutoff “e-value” of <1e-5. Domain-based comparisons with Pfam (Protein family) and KOG (a eukaryote-specific version of the Clusters of eukaryotic Ortholog Groups) databases were performed by RPS-BLAST tool from locally installed NCBI BLAST + v2.2.28 and HMMER 3.0 program, respectively. Annotated transcripts were analyzed to gene ontology (GO) classification with the aid of Blast2Go program^78^. These gene terms were then enriched on the three GO categories (Biological Process, Cellular Component and Molecular Function at level 2) using the GOseq R package^79^. Kyoto Encyclopedia of Genes and Genomes (KEGG), which is a database of biological systems, maps were retrieved by online KEGG Automatic Annotation Server for the overview of metabolic pathway analysis^17,19,20,80^.

### Differential gene expression analysis

The reads of each library were separately mapped to the *de novo* assembled transcripts with the aid of bowtie 2 program with no mismatch^81^. Count numbers of mapped reads and FPKM (expected number of Fragment Per Kilobase of transcript sequence per Millions base pairs sequenced) were retrieved and normalized by RSEM V1.2.15^82^. Differential expression statistical analysis of three treatment (NT, LT and HT) was conducted by the DEGSeq R package^83^ with a cutoff “q-value” of 0.05 and |log2(fold change)| > 1. Transcripts with absolute fold change values over 2.0 were marked as significantly differential expressed genes.

### Experimental validation by quantitative real-time PCR

11 differentially expressed genes were randomly selected for validation using quantitative real-time PCR (qRT-PCR) with gene specific primers designed using Primer 5 software (Premier Biosoft International) to validate our Illumina sequencing data. Primers were listed in Supplement 6. Samples were generated from NT, LT and HT groups in the preceding experiment. The first strand cDNA was synthesized by using M-MLV Reverse Transcription Kit (Promega, USA) from 1 μg of RNA. All the cDNA products were diluted to 500 ng/μl. The 20 μl qRT-PCR reaction mixture consisted of 2 μl cDNA template, 0.4 μl of both primer, 10 μl of KAPA SYBR®FAST qPCR Master Mix (2×), 0.4 μl of ROX and 6.8 μl of RNAase-free water. PCR amplification was performed as that incubated in a 96-well optical plate at 95 °C for 30 s, followed by 40 cycles of 95 °C for 5 s, 58 °C for 30 s, and a final extension at 72 °C for 2 min. qRT-PCR was performed using the StepOne Plus Real-Time PCR system (Applied Biosystems) and 2-ΔΔCT method was used to analysis the expression level of genes. 18S ribosomal RNA (18S) and were used as the reference gene for qRT-PCR normalization.

## Electronic supplementary material

supplement 1

supplement 2

supplement 4

supplement 5

supplement 6
