# Supplementary material for: Deep Transcriptomic Analysis of Black Rockfish (Sebastes schlegelii) Provides New Insights on Responses to Acute Temperature Stress
Source: Sci Rep. 2018 Jun 14;8:9113. doi: 10.1038/s41598-018-27013-z (PMC6002380; doi:10.1038/s41598-018-27013-z)
Supplement: Supplementary file 1 — supplement 1 [file 41598_2018_27013_MOESM1_ESM.pdf]

Title: Deep Transcriptomic Analysis of Black Rockfish (Sebastes schlegelii) Provides New Insights on Responses to Acute Temperature Stress

Author: Likang Lyu, Haishen Wen*, Yun Li*, Jifang Li, Ji Zhao, Simin Zhang, Min Song, Xiaojie Wang
Institution: The Key Laboratory of Mariculture (Ocean University of China), Ministry of Education, Ocean University of China, Qingdao, P. R. China
* The corresponding Author.
Haishen Wen, professor
Yun Li, associate professor
Tel: +86-532-82031825
E-mail: Haishen Wen (wenhaishen@ouc.edu.cn) and Yun Li (yunli0116@ouc.edu.cn)

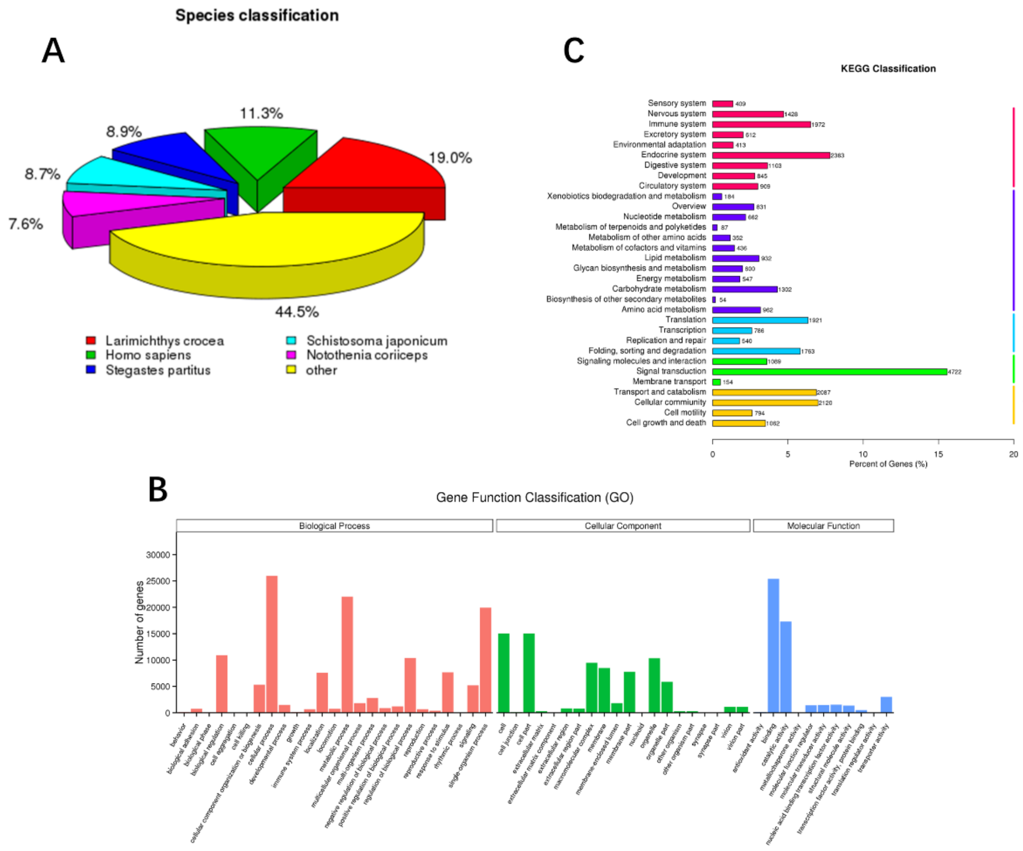

Supplement 1. Annotation and functional classification of transcripts in liver of black rockfish. (a) Top-hit species distribution of BLASTX matches of assembled transcripts. (b) Function annotation of assembled transcripts based on gene ontology (GO) analysis. GO analysis was performed at level 2 for the three main categories (biological process, cellular component and molecular function). The x-axis shows the specific terms. The y-axis shows the number of transcripts in each term. (c) Pathway assignment based on the Kyoto Encyclopedia of Genes and Genomes (KEGG) database. Transcripts were classified into five main categories (A: cellular process, B: environmental information processing, C: genetic information processing, D: metabolism and E: organismal systems).

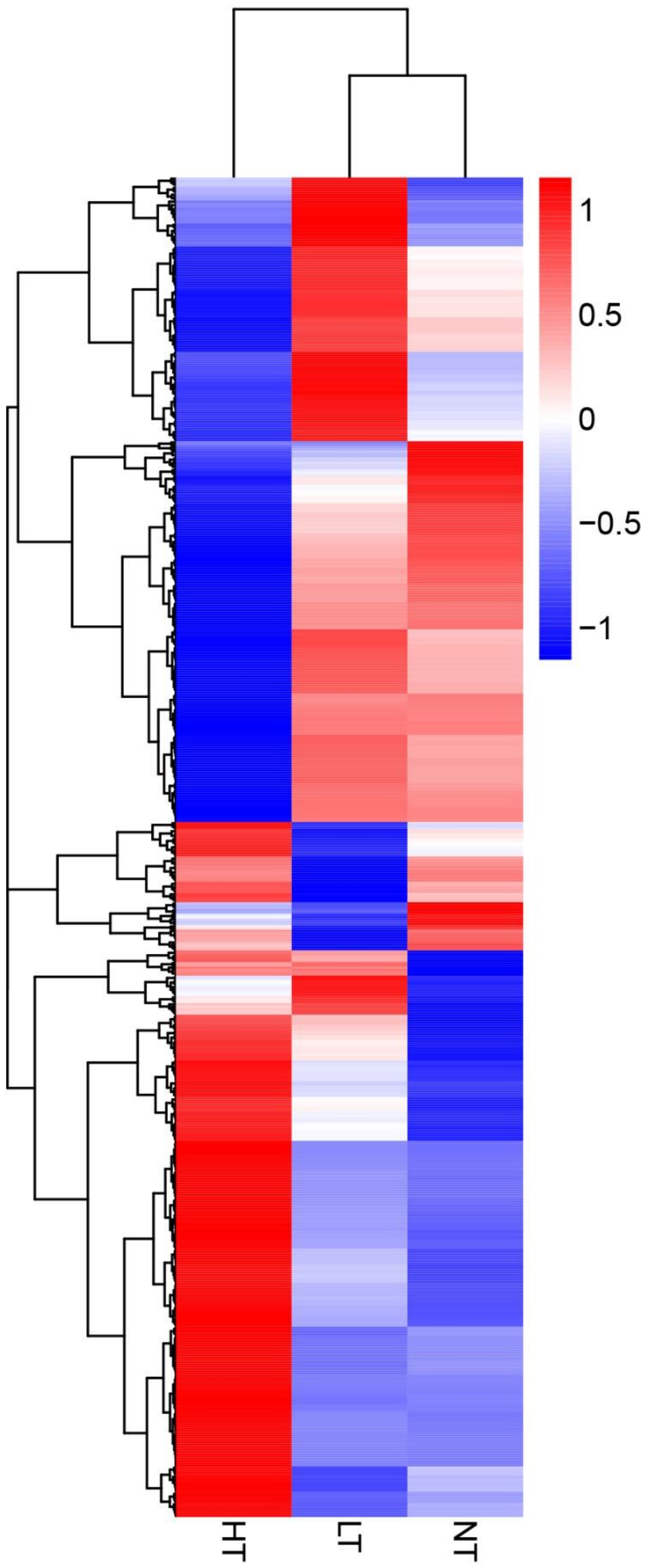

Supplement 3. Expression values of 3 libraries (HT, LT, NT) are presented in heat map as RPKM normalized log2 transformed counts. Red and blue colors indicate up- and down- regulated transcripts, respectively.
